# Sativex-induced neurobehavioral effects: causal or concausal? A practical advice!

**DOI:** 10.1186/s40199-015-0109-6

**Published:** 2015-04-17

**Authors:** Margherita Russo, Carmela Rifici, Edoardo Sessa, Giangaetano D’Aleo, Placido Bramanti, Rocco Salvatore Calabrò

**Affiliations:** IRCCS Centro Neurolesi “Bonino-Pulejo”, Contrada Casazza, 98124 Messina, Italy

## Abstract

Nabiximols (Sativex) is an oromucosal spray, containing delta-9 tetrahydrocannabinol (THC) and cannabidiol (CBD), used as treatment for unresponsive spasticity in multiple sclerosis (MS) patients. Sativex is thought to not affect cognition or induce any psychiatric problem at the doses generally used. Nonetheless, it is known that the concomitant use of more than one muscle-relaxant drugs can result in additive neuropsychiatric effects. Herein we describe a case of a woman affected by MS and treated with baclofen and methylprednisolone, who developed important behavioral changes, including suicidal ideation, after 4 weeks of Sativex administration. We are not completely able to state if Sativex alone was responsible for our patient’s psychiatric symptoms, in reason of the concomitant use of the other drugs.

In conclusion, physicians should pay more attention when prescribing drugs to MS patients affected by spasticity, including Sativex, since neurobehavioral side effects may emerge especially in predisposed individuals.

## Background

Nabiximols (Sativex) is an oromucosal spray, containing delta-9 tetrahydrocannabinol (THC) and cannabidiol (CBD), and commonly used as an add-on treatment for unresponsive spasticity in multiple sclerosis (MS) patients. Sativex common side effects include dizziness and tiredness, whereas psychiatric symptoms, such as anxiety, changes in mood, and paranoid ideas, have been rarely reported [1]. However, at the doses generally used, Sativex do not affect driving ability or have any significant detrimental effects on cognition and mood, also in aged people, as demonstrated by Rekand [2]. Nonetheless, it is known that the concomitant use of more than one musclerelaxant drugs can result in additive neuropsychiatric effects [3]. Indeed baclofen, one of the most used myorelaxants, may induce numerous neuropsychiatric adverse drug reactions, including behavioral disinhibition [4]. Moreover, corticosteroids are used in MS for the management of acute exacerbations because of their potential activity in reducing inflammation-related damage in the central nervous system (CNS). Nonetheless, methylprednisolone, a frequently used corticosteroid, may produce neuropsychiatric effects.

## Case presentation

A 55-year-old female, affected since ten years by relapsing remitting MS, according to McDonald 2001 criteria, and treated with beta-interferon (INF-β) since 2005, comes to our observation for a clinical relapse characterized by ataxia and urinary urgency. In addition, the patient presented a moderate to severe spasticity partially responsive to baclofen 50 mg/daily. Family and personal history was unremarkable, although since adolescence she reported anxiety with sporadic episodes of depressive mood. Nevertheless, since she was not diagnosed with any psychiatric illness, including major depression or bipolar disorders, she has never been treated with any specific psychoactive drugs. Physical examination was negative, whereas neurological examination showed mild ataxia, moderate to severe right hemiparesis with spastic hypertonia and hypoesthesia, bilateral dysmetria, tetrahyperreflexia, and urinary urgency, with an Expanded Disability Scale of 6, a Modified Ashwort (MAS) of 3+, Numeric Rating Scale for Spasticity (NRS) of 10, Ambulation Index (AI) of 5. Hematochemical tests, including autoimmune and thyroid screening, were normal. A brain and spinal cord MRI showed multiple T2 weighted hyperintensities, mainly involving the periventricular and juxtacortical areas, and brainstem, with an enhancing-lesion in the cerebellum. Methylprednisolone 8 mg/daily for 3 days, followed by 4 mg/ daily for other 3 days, was thus prescribed, with a significant improvement in urinary urgency and ataxia, after around a week. Moreover, she was also prescribed Sativex, according to our regional (Sicily) criteria, to improve her severe spasticity, till reaching a daily dosage of ten puffs (2,7 mg of THC and 2,5 mg of CBD *per* single-dose). At one month-follow-up she had a good response to the treatment, as showed by nearly all the clinical scales (MAS 3-, NRS 6, AI 5). However, his husband referred that she presented important behavioral changes with alternations of maniac and depressive phases, and suicidal ideation, so to suggest a substance induced mood disorder. Nabiximol was thus stopped with a complete remission of psychiatric symptoms within 10 days. During such period no specific psychiatric drugs were needed, but only low dose benzodiazepine (0,50 mg alprazolam) to overcome her insomnia. At 3 month-follow-up she did not present any psychiatric problem.

## Discussion and conclusions

The “probable” role of Sativex in the occurrence of the psychotic symptoms presented by our patient is demonstrated by the fact that symptoms appeared after Nabiximol was introduced, and disappeared after Sativex discontinuation. A total score of 5 (probable) was obtained on the Naranjo causality scale. The CNS side effects of Nabiximols are generally mild to moderate, are usually well tolerated, and tend to disappear with continued therapy, or with reduction of initial dosage. Nevertheless, hallucinations, delusional beliefs or transient psychotic reactions have also been reported. The brain endocannabinoid (eCB) system modulates several neurobiological processes and its dysfunction is suggested to be involved in the pathophysiology of mood and drug use disorders. The psychiatric disorders related to cannabis use are mainly anxiety and depressive mood, although cannabis use has been linked with the neurobiology of schizophrenia. Indeed, the CB1 receptor-mediated signaling has been shown to play a critical role in the neural circuitry mediating mood, motivation, and emotional behaviors. Moreover, an increase in CB1 receptor levels is associated with suicide.

Although data on the role of eCB system in the pathophysiology of major depression and suicide have been reported, it remains to be clearly understood whether alterations in the CB1 receptor-mediated signaling in selective brain regions are linked to neurobehavioral disorders, including depressive mood [5].

Moreover, a possible role of the GABA transmission in the pathophysiology of mood disorders may also be hypothesizable, since Sativex also act on GABA-B receptors, which in turn have been demonstrated to modulate serotoninergic neurotransmission.

However, we are not completely able to state if Sativex alone was responsible for our patient’s psychiatric symptoms, as they could be also related to the concomitant use of baclofen and methylprednisolone. Indeed, baclofen could have a complex and indefinite action on the dopaminergic or serotoninergic system, through which mania may be induced in vulnerable subjects. Notably, many other agents, including corticosteroids, have the potential for inducing behavioral side effects.

Emerging evidence have also demonstrated a genetic contributions to the depressive disorders through family studies and molecular genetics, with specific additional events (as a new drug introduction) precipitating depressive illness in such predisposed individuals [6].

In conclusion, physicians should pay more attention when prescribing drugs to MS patients affected by spasticity, including Sativex, since neurobehavioral side effects may emerge especially in predisposed individuals.

## Consent

Written informed consent was obtained from the patient for the publication of this report and any accompanying images.

## Abbreviations

AI: Ambulation index
INF-β: Beta-interferon
eCB: Brain endocannabinoid system
CBD: Cannabidiol
CNS: Central nervous system
THC: Delta-9 tetrahydrocannabinol
GABA: Gamma-amino-butirric-acid
MS: Multiple sclerosis
MAS: Modified Ashwort
NRS: Numeric rating scale for spasticity
